# DACH1 is a novel predictive and prognostic biomarker in hepatocellular carcinoma as a negative regulator of Wnt/β-catenin signaling

**DOI:** 10.18632/oncotarget.3281

**Published:** 2015-04-02

**Authors:** Yu Liu, Rong Zhou, Xun Yuan, Na Han, Si Zhou, Hanxiao Xu, Mingzhou Guo, Shiying Yu, Cuntai Zhang, Tiejun Yin, Kongming Wu

**Affiliations:** ^1^ Department of Geriatrics, Tongji Hospital of Tongji Medical College, Huazhong University of Science and Technology, Wuhan, P.R. China; ^2^ Department of Oncology, Tongji Hospital of Tongji Medical College, Huazhong University of Science and Technology, Wuhan, P.R. China; ^3^ Department of Gastroenterology and Hepatology, Chinese PLA General Hospital, Beijing, P.R. China

**Keywords:** DACH1, hepatocellular carcinoma, Wnt/β-catenin signaling, prognostic factor, TMA

## Abstract

The cell fate determination factor Dachshund (DACH1) functions as a novel suppressor in the progression of various neoplasms. Previous study has suggested that hypermethylation of promoter region was responsible for the reduction of DACH1 expression in hepatocellular carcinoma (HCC), and associated with the progression of HCC, but the clinical significance and the exact molecular mechanisms of DACH1 in the progression of HCC remain unclear. In this study, we employed public microarray data analysis and tissue microarrays (TMAs) technologies and showed that DACH1 expression was reduced in HCC even at early stage and associated with the tumor progression. Notably, Kaplan-Meier analysis further indicated DACH1 could be an independent prognostic factor for the overall survival of HCC. Further, mechanistic studies revealed that overexpression of DACH1 inhibited the growth and migration of HCC cell line, which were dependent in part on the inactivation of Wnt pathway via phosphorylation of GSK3β to suppress β-catenin. In agreement, the abundance of DACH1 was inversely correlated with several Wnt target genes. Collectively, our study indicated β-catenin is a novel target of DACH1 in HCC.

## INTRODUCTION

Despite significant achievement has been made in early diagnosis and surgical intervention, hepatocellular carcinoma (HCC) still ranks the leading cause of cancer-related mortality, with < 26% patients surviving beyond 5 years [1]. As is true in most disease processes, the best chance for long time survival of HCC comes from the active surveillance of patients known to be at high risk so that they can be diagnosed and treated at early stage [2]. At present, the serum α-fetoprotein (AFP) has been utilized extensively to screen underlying HCC; however, normal AFP levels are present in as many as 30% of patients at the time of diagnosis and usually remain low expression even with advanced HCC [3]. There are no effective clues for screening AFP negative HCC patients. Thus, it is imperative to identify the early stage HCC biomarkers to improve diagnostic methods. In addition, identification of biomarkers could improve our understanding of cellular and molecular mechanisms engaged in the pathogenesis of HCC and provide new targets for drug development and gene therapy [4].

The mammalian DACH1 is a key component of the retinal determination gene network (RDGN) family, which was originally classified exclusively as the primary administrator in organismal determination in the *drosophila eye* but is now described to carry out a variety of different functions in tumorigenesis and metastasis [5–7]. In this conserved and sophisticated network, DACH1 operates ordinarily via interacting with DNA-binding transcription factors (c-Jun, Smads, Six, ERα) [8–12] or directly be recruited into specific DNA sequence [13]. Several lines of evidence have indicated the potential value of DACH1 as a prognostic factor in breast cancer for its strong connection between the poor clinical outcome and lower expression of DACH1 [11, 14, 15]. Of note, much attention has been focused on the ability of DACH1 to repress the process of Epithelial-Mesenchymal Transition (EMT) and to reduce the subpopulation of cancer stem cell (CSC)[15, 16], supporting its role as a novel tumor suppressor.

Current evidence indicates aberrant expressions of multiple signaling pathways ignite the initiation of hepatocarcinogenesis and even far accelerate the process of metastasis [17]. Termination of hepatocyte proliferation at G1 phase and protective response to p53-dependent cell apoptosis, which is guided by TGF-β pathway, are crucial negative feedback mechanisms in normal regeneration [18]. The loss of anti-proliferative function induced by TGF-β/Smad signaling might be a decisive factor in the rapid intrahepatic metastasis. Intriguingly, our previous study demonstrated that methylation silence in the promoter region of DACH1was significantly associated with resistance to such anti-proliferative effect in HCC. Contrarily, overexpression of DACH1 could activate TGF-β1 expression and increase the chemosensitivity to 5-fluorouracil (5-FU) in HCC [19]. Meanwhile, epigenetically silenced expression of DACH1 in several kinds of cancers could be restored by HDACs inhibitors and resulted in reduced proliferation [19, 20], thus providing a potential approach by the demethylase treatment to recover the expression of DACH1 in HCC patients.

Most HCCs are a continuous process starting from hepatitis, cirrhosis, hyperplasia, and finally progress to cancer. To further understand the role of DACH1 in hepatocarcinogenesis and evaluate the prognostic value of DACH1 in HCC, we performed a combined analysis of publicly available microarray data and tissue microarrays (TMAs). Furthermore, a series of experiments in human cell line of HCC were conducted to investigate the effect of DACH1 on cell growth and invasion *in vitro*. Our study indicated that DACH1 was specifically reduced in HCC in comparison with liver benign diseases and significantly correlated with clinical outcome. In addition, we found β-catenin was a novel target of DACH1 in HCC.

## RESULTS

### Downregulation of DACH1 expression in HCC patients

**Figure 1:**
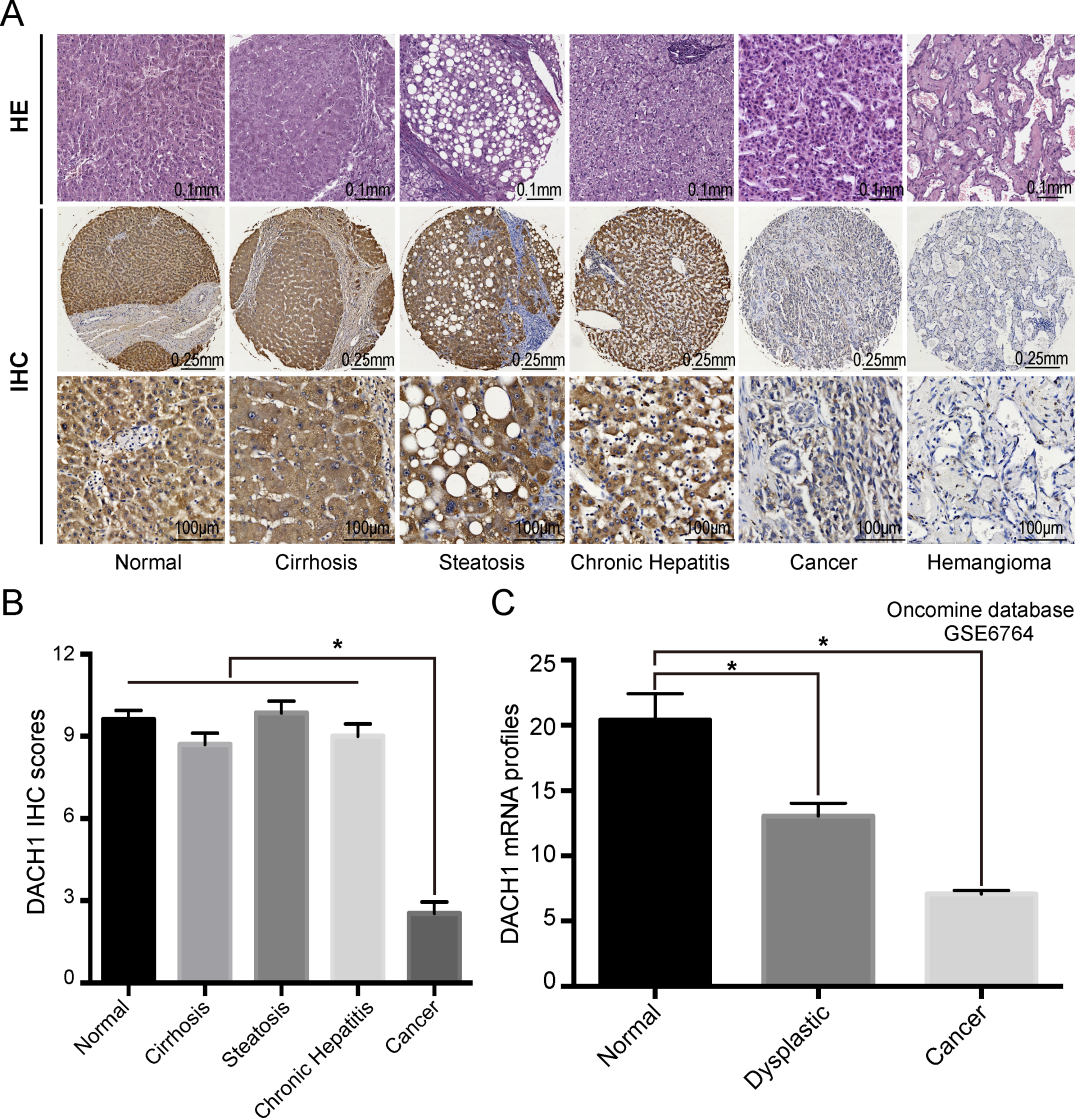
Combined analyses of DACH1 in liver benign and malignant lesions Representative images of DACH1 expression in different lesions of liver were shown **(A)** with semi quantitative result displayed as mean ± SE **(B)**. Oncomine database analysis showed the mRNA level of DACH1 in normal and cancer tissues **(C)**.

In order to comprehend the significance of DACH1 expression in liver benign as well as malignant lesions, we analyzed DACH1 protein levels in a TMA containing 120 informative patients with various hepatic lesions, which mainly included cirrhosis, steatosis, chronic hepatitis, hemangioma and cancer. DACH1 protein was predominantly detected in the cytoplasm of hepatocyte, and representative images of immunohistochemical staining for noncancerous and cancerous tissue are shown in Fig. 1A. Tissues from hepatic hemangioma patients were DACH1 negative (Fig. 1A), indicating DACH1 specifically expressed in hepatocyte. Next, we examined the potential association of DACH1 expression with the clinicopathological parameters by using semi-quantitative criteria. No statistical significance was found for DACH1 expression in cirrhosis, steatosis, chronic hepatitis and normal tissue (*f* test, *p* = 0.194). However, DACH1 protein levels were considerably decreased in primary HCC (*t* test, *p* < 0.001) (Fig. 1B).

To evaluate whether the mRNA expression of DACH1 is consistent with the protein abundance, Oncomine database (GSE6764) was interrogated. The relative abundance of DACH1 was reduced when the normal tissue transformed into dysplastic phenotype (*t* test, *p* = 0.006). DACH1 mRNA was decreased 2- to 3-folds in HCC compared to normal liver (*t* test, *p* < 0.001) (Fig. 1C). Our results suggested that downregulation of both mRNA and protein levels of DACH1 occurred in HCC patients when compared to normal liver or other benign lesions.

### Low levels of DACH1 significantly correlate with progression of human HCC

**Figure 2:**
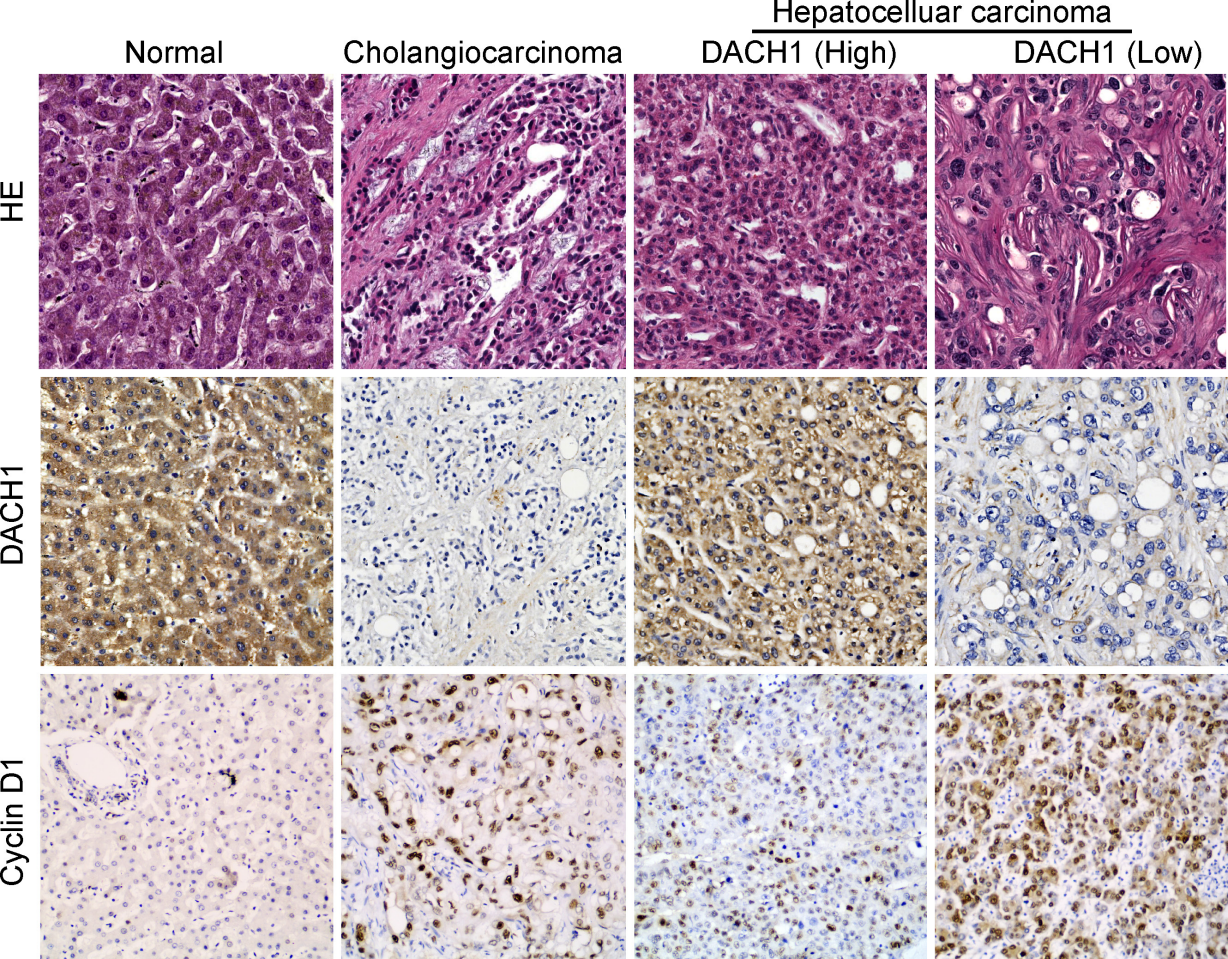
Immunohistochemistry analysis of DACH1 and cyclin D1 on HCC tissues In consecutive TMA slides, the representative HE staining and immunohistochemistry images presented for DACH1 and cyclin D1.

To further explore the role of DACH1 in HCC development, we employed a larger series of HCC patients. The microarray included more HCC patients with various tumor grades and stages (*n* = 95) and additional control groups (e. g., benign inflammation and normal tissue). DACH1 expression in tumor tissues was arbitrarily classified as high (IHC score ≥ 6) in 48 cases and low (IHC score < 6) in 47 HCC patients. We also investigated the expression of cyclin D1, because it is a crucial proliferate hallmark of multiple cancers and previous reports confirmed cyclin D1 gene was a functional target of DACH1 [11, 20, 21]. Immunohistochemical staining confirmed that DACH1expression was low in cholangiocarcinoma and primary HCC (Fig. 2). Intriguingly, expression of DACH1 was strongly correlated with the tumor progression. In the low DACH1 group, the expression level of cyclin D1 was significantly higher than it in the high group (Fig. 2). In comparison with the clinicopathological parameters between groups, including age, sex, tumor grading, TNM staging as well as the expression of cyclin D1, we found the level of DACH1 expression was significantly associated with tumor grade (χ^2^ test, *p* < 0.001) and illustrated a reverse coordination with cyclin D1 (χ^2^ test, *p* = 0.048), whereas age, sex and TNM staging showed no-statistical significance with DACH1 (Table 1).

**Table 1:** Correlations between DACH1 and clinicopathological features of 95 HCC patients in TMA (LV1201)

Variables	DACH1 staining		*P* value
	≥ 6 (high expression)	6 < (low expression)	
Age	50.73 ± 1.83	50.45 ± 1.54	0.907^a^
Sex			0.130^b^
Male	40	33	
Female	8	14	
Grade			< 0.001^b^
1 + 2	44	24	
3	4	23	
TNM			0.081^b^
I + II	28	19	
III + IV	20	28	
Cyclin D1			0.048^b^
Positive	22	31	
Negative	26	16	

a Independent-Sample *T* test

b Person Chi-square test.

**Figure 3:**
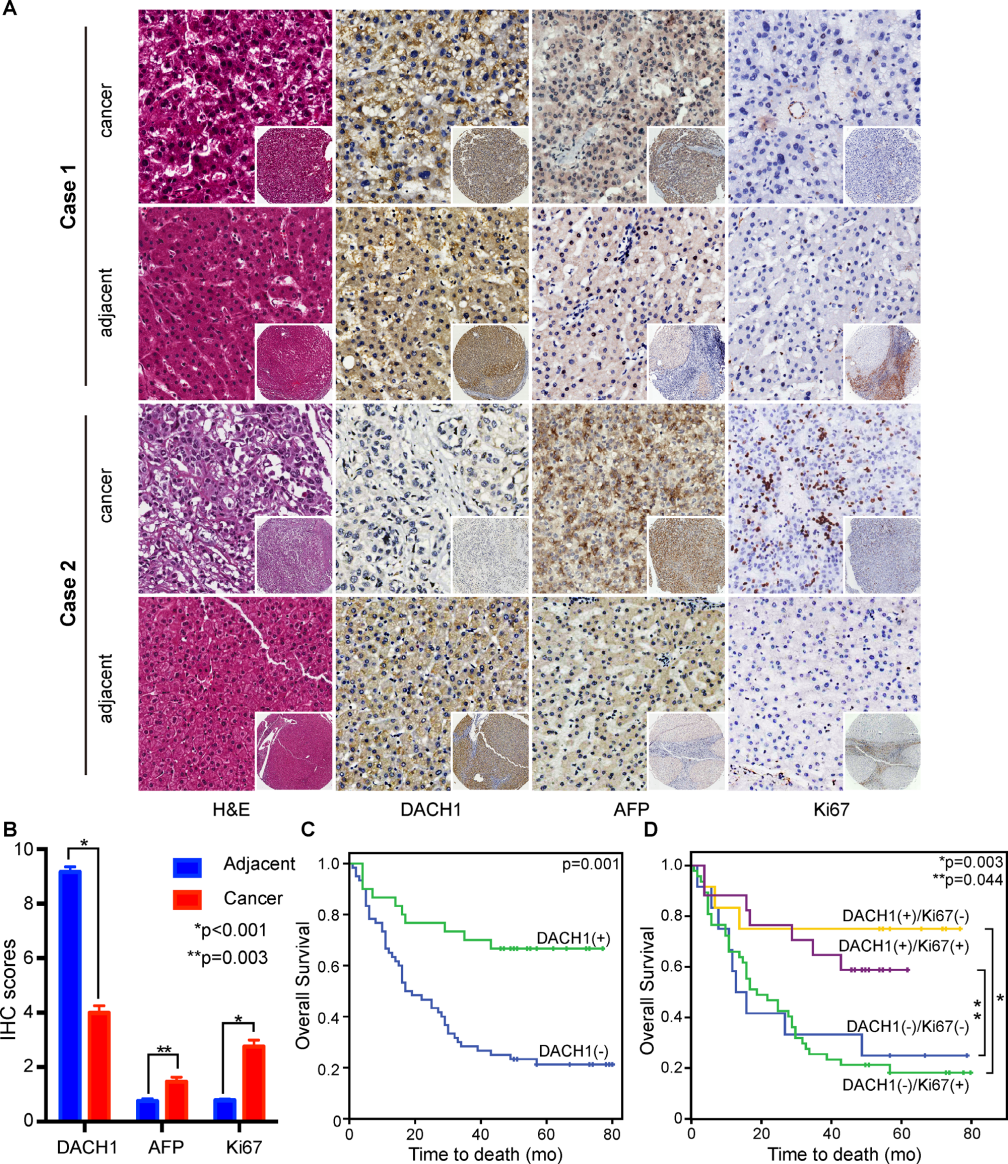
DACH1 is an independent prognostic factor of overall survival in HCC patients In a seven years cohort, representative images of the cancerous and adjacent tissue from the longest survival patient and the shortest one were shown by using HE and IHC staining **(A)**. The quantification of data was displayed as mean ± SE **(B)**. Kaplan-Meier survival curve of DACH1 alone **(C)** or combined with Ki-67 **(D)** were analyzed.

To understand the value of DACH1 in the prediction of HCCs’ prognosis, we examined expression of DACH1 in different tissue microarray containing 90 pairs of HCC samples, which provided survival data and detailed information about AFP and Ki-67 expression. The profiles of DACH1, AFP and Ki-67 of two representative cases were shown in Fig. 3A, Case1 had the longest survival and case 2 had shortest survival in this cohort. In comparison of adjacent tissues, the majority of HCC cancerous samples showed drastic reduction of DACH1 and increased expression of AFP and Ki-67 in this seven years cohort (Fig. 3B). Segregation of these patients into DACH1-high and low expression groups did not reveal significant relationship with clinicopathological parameters of sex, age, liver cirrhosis, tumor size, location, pathological type or the expressions of AFP, but noticeably associated with tumor grading (*p* = 0.033), TNM staging (*p* = 0.037) and Ki-67 profiles (*p* = 0.037) (Table 2). Median overall survival time of the DACH1-high and DACH1-low cases was 29 and 17 months, respectively, indicating significant difference of survival (Kaplan Meier log-rank test, *p* = 0.001, Fig. 3C). As expected, low expression of Ki-67 tended to predict better clinical outcome, but it did not reach the statistical significance in part for the limited sample size and variation of data (Kaplan Meier log-rank test, *p* = 0.170). Combined examination of DACH1 with Ki-67 would provide more precise information for the clinical outcome of HCC patients. Apparently, patients characterized by high levels of DACH1 and negative Ki-67 index had the longest OS in this study. However, there was no statistical significance between the DACH1-/Ki-67+ and DACH-/Ki-67− patients (Fig. 3D).

**Table 2:** Profiles of patients with DACH1-high expression or DACH1-low expression in TMA (HLiv-HCC180Sur-02)

Variables	DACH1 expression		*P* value
	6 < (low expression)	≥ 6 (high expression)	
Age			0.511^a^
< 50	16	10	
50 ≥	44	20	
Sex			1.000^b^
Male	52	26	
Female	8	4	
Liver cirrhosis			
Absent	39	19	0.876^a^
Present	21	11	
Maximal tumor size			
≤ 5 cm	20	16	0.068^a^
> 5 cm	40	14	
Tumor location			0.712^a^
Left	6	4	
Right	16	12	
Others	28	14	
Pathological type			0.778^a^
Nodular type	28	16	
Giant mass type	18	7	
Others	14	7	
Grading			
1~2	38	24	0.033^a^
3~4	28	6	
TNM staging			0.037^a^
I~II	24	19	
III~IV	36	11	
AFP			0.527^a^
Low expression	36	20	
High expression	22	9	
Ki-67			0.037^a^
Negative	12	12	
Positive	47	17	

a Person Chi-square test

b Continuity correction Chi-square test

We also investigated the association between cumulative overall survival-rates and clinicopathological factors by univariate Cox regression analysis. As shown in Table 3, the expression of DACH1 protein (HR = 2.806; 95% CI, 1.318 ~ 5.974; *p* = 0.007) and TNM stage (HR = 0.420; 95% CI, 0.195 ~ 0.905; *p* = 0.027) were prognostic factors for OS, whereas other clinicopathological factors were not directly related to the clinical outcome of HCCs. We performed the forward variable-selection procedure using the two factors and the DACH1 expression was identified as an independent predictive factor for the overall survival for HCC patients (HR = 2.984; 95% CI, 1.489 ~ 5.982; *p* = 0.002).

**Table 3:** DACH1 expression in HCC is an independent prognostic factor for HCC patients (HLiv-HCC180Sur-02)

Variables	Univariate Analysis		Variable Selection	
	HR (95%CI)	*P* Value	HR (95%CI)	*P* Value
Age	0.994 (0.964~1.024)	0.687		
Sex (Male vs. Female)	0.841 (0.351~2.013)	0.698		
Liver cirrhosis (Absent vs. Present)	0.777 (0.411~1.471)	0.439		
Maximal tumor size (≤ 5 cm vs. > 5 cm)	0.582 (0.275~1.233)	0.158		
Tumor location (Left vs. Right vs. Others)	0.499 (0.161~1.545) 0.701 (0.25~1.943)	0.421		
Pathological type (Nodular vs. Giant vs. Others)	1.654 (0.615~4.451) 1.633 (0.683~3.901)	0.506		
Grading (1~2 vs. 3~4)	1.189 (0.586~2.415)	0.632		
AFP expression (Low vs. High)	0.923 (0.475~1.793)	0.813		
Ki67 expression (Negative vs. Positive)	0.818 (0.397~1.686)	0.586		
DACH1 expression (Low vs. High)	2.806 (1.318~5.974)	0.007	2.984 (1.489~5.982)	0.002
TNM staging (I~IIvs. III~IV)	0.420 (0.195~0.905)	0.027	0.425 (0.241~0.749)	0.003

### Overexpression of DACH1 suppresses the proliferation and migration of HCC cell *in vitro*

**Figure 4:**
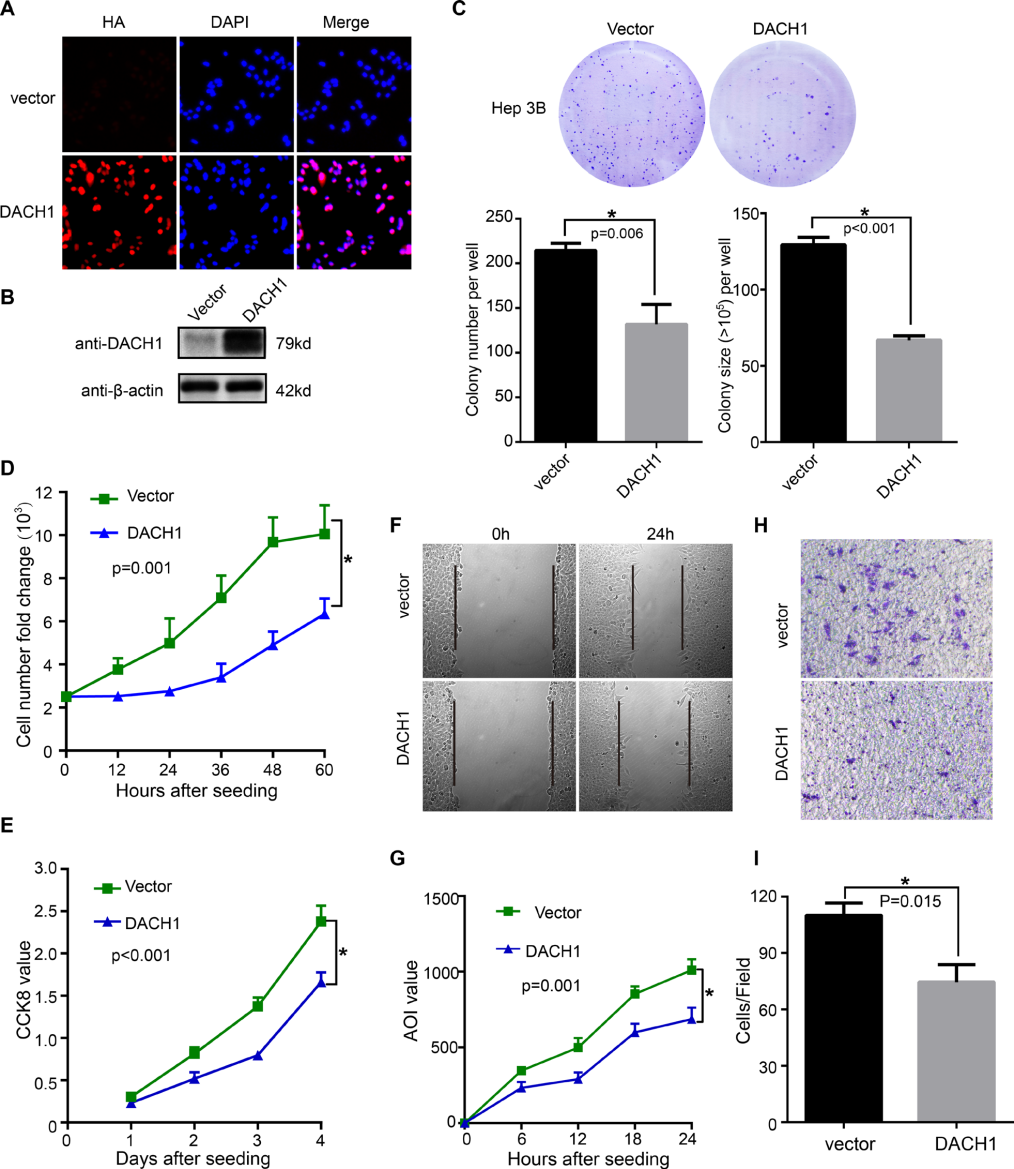
Overexpression of DACH1 suppresses cell growth and migration of HCC cell line *in vitro* Hep3B cells stably expressing DACH1 were confirmed by **(A)** immunofluorescent stain and **(B)** Western blotting. Representative examples of colonies and quantification of data were shown as mean ± SE **(C)**. Cellular proliferation was measured by cell number counting **(D)** and CCK-8 value **(E)**, with the data displayed as mean ± SE. Hep3B cells were assessed for migration ability by wound closure **(F)** and the data shown as mean ± SE **(G)**. Transwell assays were employed to examine the invasion **(H)** with results shown as mean ± SE **(I)**.

To investigate the role of DACH1 in tumor biological behaviors, we generated HCC cell line that stably expressing DACH1. Expression of DACH1 was confirmed by immunofluorescent assay (Fig. 4A) and Western blot (Fig. 4B). Subsequently, we used this stable cell lines to evaluate the role of DACH1 in cell proliferation and migration of HCC.

To explore the significance of DACH1 on cell growth, Hep3B cells expressing DACH1 or vector control were evaluated by colony formation, cell number counting and cell counting kit-8 (CCK8) assay. The colony number and size were decreased by DACH1 in Hep3B cell lines (Fig. 4C), which were also supported by the results of cell counting (Fig. 4D) and CCK8 staining (Fig. 4E). These results indicated that DACH1 could repress HCC cellular growth and proliferation *in vitro*.

To determine whether overexpression of DACH1 had a crucial role in migration and invasion, we performed scratch wound test and Transwell assay. Our results demonstrated a noticeable reduction in migration ability of HCC cells expressing DACH1, and a slower closure in DACH1-Hep3B cells (Fig. 4F and 4G). The number of invaded cells significantly decreased in HCC cells expressing DAHC1 in comparison with the GFP vector cells (Fig. 4H and 4I), indicating that the ectopic expression of DACH1 markedly suppressed the invasive ability of HCC cells.

### DACH1 inhibited HCC growth through suppression of Wnt/β-catenin signaling

**Figure 5:**
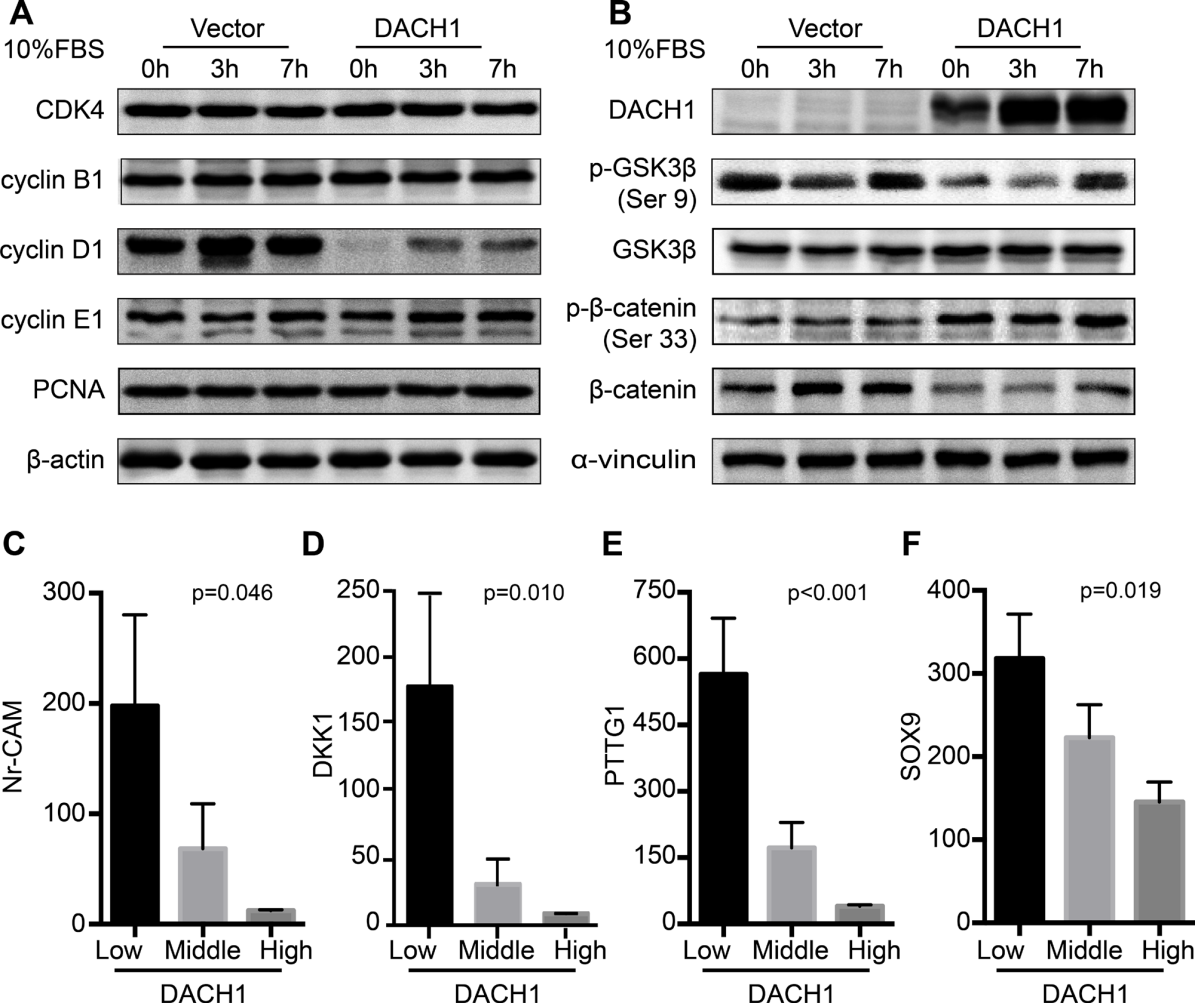
DACH1 suppresses the activation of Wnt/β-catenin signaling Western blot to evaluate cell cycle related protein expressions **(A)** and protein abundance of Wnt/β-catenin pathway **(B)**. Analysis of GEO database shows correlation between DACH1 and β-catenin downstream targets Nr-CAM **(C)**, DKK1 **(D)**, PTTG1 **(E)** and SOX9 **(F)** by one-way ANOVA test.

Cell cycle proteins regulated by DACH1 might be responsible for the inhibited proliferation in HCC. To address the underlying mechanisms, protein levels of cell cycle elements were screened by western blot. Cells were serum starved for 48 h and supplemented with 10% fetal bovine serum to activate cells to enter the cell cycle, and cells were collected in 0h, 3h and 7h, respectively. Under the basal condition, DACH1 significantly suppressed the abundance of cyclin D1. Serum stimulation induced significant expression of cyclin D1 in vector group, whereas overexpression of DACH1 markedly diminished the activation of cyclin D1 (Fig. 5A). However, there was no statistical significance observed with DACH1 overexpression and the protein abundances of CDK4, cyclin B1, cyclin E1 and PCNA. Therefore, these results suggested that decreased abundance of cyclin D1 induced by DACH1 might play a key role in negative regulation of proliferation and cell-cycle progression in HCC.

Since the cyclin D1 is one of the major downstream targets of Wnt/β-catenin signaling and the DACH1 is associated with Wnt pathways in colorectal cancer [20], we hypothesized that DACH1 could also repress cyclin D1 abundance via inactivation of Wnt signaling in HCC. To test whether DACH1 affected the activation of Wnt/β-catenin signaling, we measured the protein level of β-catenin, phosphorylation of β-catenin (Ser 33), glycogen synthase kinase-3β (GSK3β) and phosphorylation of GSK3β (Ser 9) by western blot. Ectopic expression of DACH1 significantly inhibited the p-GSK3β levels without affecting total GSK3β. The abundance of phosphorylated β-catenin was upregulated in DACH1-overexpression group. In agreement, the β-catenin level was decreased (Fig. 5B). Thus, these findings suggest that DACH1 could suppress the hepatocellular carcinoma growth through inhibition on Wnt signaling, which might be mediated by phosphorylation of GSK3β to suppress the activation of β-catenin.

In order to further investigate the relationship between DACH1 and Wnt signaling at mRNA level, we analyzed public microarray dataset of GSE6764, which was performed to identify the gene-expression profiles of the 20 noncancerous tissues, 15 dysplastic liver tissue and 37 samples from different phases of primary HCC. The known downstream targets of β-catenin, Neuron-glial related cell adhesion molecule (Nr-CAM), Dickkopf-1 (DKK1), Pituitary tumor-transforming gene 1 (PTTG1) and SOX9, were examined to identify the relationship with DACH1 mRNA profiles. Based on the level of DACH1 mRNA profiles, the group has been divided into three parts: low-level (0% ~ 33%), middle-level (34% ~ 66%) and high-level (67% ~ 100%). We examined the correlations of DACH1 with known β-catenin downstream targets and found that increased expression of DACH1 expression significantly downregulated expression of Nr-CAM (Fig. 5C), DKK1 (Fig. 5D), PTTG1 (Fig. 5E) and SOX9 (Fig. 5F). Thus, the results indicated that DACH1 was inversely correlated with β-catenin downstream targets in HCC.

## DISCUSSION

Overwhelming evidence has proved that normal embryogenesis and neoplasia share many basic processes and molecular pathways. Tumorigenesis may derive from the loss control of normal developmental gene [22]. For example, abnormal functions of conserved RDGN play critical roles in tumor initiation and progression [5–7]. Specifically, DACH1 restrains the tumorigenesis as well as influences the malignant phenotype of carcinoma, mainly including breast cancer [11, 16, 23], renal cancer [21], lung [24], colorectal [20] and prostate cancer [10]. This paper provided results that DACH1 was lost in HCC and associated with the tumor progression as recently published [19]. Our study further indicated DACH1 was an independent prognostic factor for the postoperative HCC patients. Additionally, ectopic expression of DACH1 reduced the growth and migration of HCC cells *in vitro*, which may depend on the inactivation of Wnt pathway via phosphorylation of GSK3β to suppress the activation of β-catenin. Thus, we concluded that endogenous DACH1 plays a protective role in the progression of HCC through suppression of Wnt/β-catenin pathway.

Over the past few decades, extensive studies have identified the fundamental pathways, which are responsible for regulating cell determination and apoptosis, associated with the development of liver cancer [25]. Given the role of Wnt/β-catenin pathway in specifying cellular fates during both embryonic development and adult tissue regeneration, it is not astonished that aberrant activation of Wnt/β-catenin signaling occurs in one third of all HCCs, emphasizing the importance of this pathway in hepatocarcinogenesis [26]. In the canonical Wnt signaling, the key transduction partner, β-catenin, is phosphorylated at Ser37 and Ser33 by a multiprotein complex composed of the adenomatous polyposis coli (APC), Axin, and GSK3β to keep low activity in the cytoplasm. Activation leads to disruption of such β-catenin degradation complex, resulting in accumulation of β-catenin followed by translocation into the nucleus to interact with transcriptional factors to trigger downstream targets. Ectopic expression of β-catenin target genes promotes HCC cell migration and invasion through Wnt signaling axis. A large, multicenter study demonstrated that elevated expression of DKK1 alone or combined with other factors could indicate poor prognostic outcome of HCC patients [27]. Meanwhile, many known metastasis suppressors function through inhibition on Wnt-EMT activity in the aggressiveness development of HCC [28]. Current study has proved that the increased expression and mutations of Wnt/Fizzled genes as well as decreased expression of Wnt inhibitors could lead to the activation of Wnt/β-catenin pathway [29]. However, β-catenin activation induced by crosstalk with other pathways cannot be excluded.

A novel finding of our study is that DACH1 could repress the activation of Wnt pathway by phosphorylation of GSK3β. *In vitro* experiments indicated that DACH1 decreased the phosphorylation of GSK3β, leading to an increasing of phosphorylated β-catenin, and then inactive Wnt pathways to repress cell proliferation and migration. Moreover, the public microarray dataset analysis revealed that several downstream targets of β-catenin (Nr-CAM [30], DKK1, PTTG1 [31] and SOX9 [32]) were negatively correlated with DACH1 level. These findings raise a possibility that endogens DACH1 functions as an inhibitor of the HCC cell proliferation and invasion being accompanied by reduced p- (Ser 9) GSK3β, which leads to suppression of Wnt signaling. Based on the previous report that the hypermethylation in the promoter region could give rise to loss or reduction of DACH1 in HCC [19], it is convincible that such epigenetic changes may be at least in part responsible for the aberrant activation of β-catenin downstream targets.

Although great endeavor has been placed on the exploration of the molecular mechanism in oncogenesis and metastasis *in vivo* and *in vitro*, it still needs more evidence to clarify the clinical significance of DACH1. An elegant study by using an artificial neural network (ANN) has proved that in breast cancer patients with Luminal A phenotype, low expression of DACH1 predicted shorter survival and disease free interval [14]. These findings were also corroborated by an earlier analysis of over 2100 samples, which identified the role of DACH1 as a potential predictor of survival in breast cancer [13]. Current study indicated DACH1 as a unique prognostic candidate in HCC patients and its profile independent of the AFP expression, making DACH1 as a valuable marker to screen both AFP positive and negative HCC patients.

It is generally known that primary HCC is associated with hepatitis through a stepwise and irreversible process. The successful intervention of malignancy requires sensitive and specific biomarker for the early diagnosis. Indeed, a large amount of work has been conducted to identify molecular markers to predict survival or guide a personalized therapy [33]. However, a particular difficulty is the lack of an available method for distinguishing between advanced hepatocirrhosis and HCC *in situ*. We found that DACH1 expression showed non-statistical difference in liver benign lesions compared with normal tissue. However, DACH1 expression could drastically reduce at early stage of HCC, and even dysplastic changes was tightly correlated with downregulation of DACH1, indicating the analysis of DACH1 might be useful for the potential screening for the super high-risk population of HCC by liver biopsy in patients with chronic hepatitis or other benign lesion. Previous study showed the reduction of DACH1 expression was related to an elevated AST/ALT ratio [19]. Our investigation further proved decreased DACH1 expression linked with lower tumor grading as well as staging. Furthermore, the negative relationship between Ki-67 staining and DACH1 suggests DACH1 may function against the proliferation of HCC, which is coordinated with the experimental results *in vitro*. However, DACH1 occupies a more prominent role in the prognosis of HCC patients compared with Ki-67 index, since endogenous expression of DACH1 might not only be responsible for the inhibition of malignant proliferation, but also interact with other cytokines in tumor microenvironment like IL-8 [12] or CXCL5 [34]. The next major challenge is exploring how to recover DACH1 gene expression to reverse the malignant activity of cancer cells in liver.

In conclusion, this study confirms the frequent downregulation of DACH1 in two independent, large cohorts of HCC samples based on TMAs analyses, supporting its role as a tumor suppressor as well as an independent prognostic factor of overall survival in HCC patients. Identification of the suppressive role of DACH1 on Wnt/β-catenin pathways could provide a novel insight in understanding the mechanism of HCC development and provide specific therapeutic strategies for patient treatment.

## MATERIALS AND METHODS

### HCC tissue microarray and immunohistochemistry

Commercially available tissue microarray (TMA) slides (BC03119, LV1201, US Biomax, Inc., Rockville, MD) containing histologically confirmed tissues from a variety of diseases were purchased for immunohistochemistry (IHC) analysis. Another tissue microarray (HLiv-HCC180Sur-02, Shanghai Biochip Co., Ltd., Shanghai, China) with 90 matched pairs of primary HCC samples and adjacent liver tissues was applied to evaluate the prognostic value of DACH1 based on its detailed survival data. Specific primary antibodies against DACH1 (ProteinTech Group) and cyclin D1 (Cell Signaling Technology) were used for IHC with a 2-step protocol. Whole slide image capture was performed on a Nano Zoomer 2.0 HT slide scanner (Ha mamatsu Photonics K.K., Hamamatsu, Japan) and captured by Nano Zoomer Digital Pathology view version 1.6.

### Analysis and quantification of staining

The immunohistochemical score were assessed by two experienced pathologists without knowledge of patients’ characteristics. Scores were calculated on intensity and percentage of positive staining tumor cell nuclei (cyclin D1) or cytoplasm (DACH1) in the whole tissue stains according to the Fromowitz Standard as described above [35, 36]. Briefly, the staining intensity was graded as follows: no staining, 0; weakly positive, 1; moderately positive, 2; and strongly positive, 3. The percentage of positive cells was into 4 grades: 0%–25% staining, 1; 26%–50% staining, 2; 51%–75% staining, 3; and 76%–100% staining, 4. The multiplication of the intensity and percentage scores was used to calculate the final staining score, which was then categorized as low (scores < 6) or high (scores ≥ 6). For quantification, all stains were assessed at 200×magnifications and at least 3 fields from each core were counted.

### Cells culture and transfection

Hep3B cell lines were cultured in Dulbecco's modified Eagle's medium (DMEM) supplemented with 10% fetal bovine serum (HyClone, Logan, UT). Cells were maintained in an atmosphere of 5% CO_2_ in a humidified 37°C incubator. Hep3B cells were seeded at 50% confluence in 6cm plates on the day prior to transduction. pKW10- DACH1 plasmid was a gift from Dr. Cvekl. DACH1 subcloned into lentivirus expression vector [37]. HEK 293T cells were transiently transfected with the appropriate combination of expression vectors or control vector with package plasmids by Lipofectamine™ 2000 (Invitrogen, Carlsbad CA, USA) according to the manufacturer's instructions as previously described [11]. Then the viral supernatants were harvested 48 h after transfection and filtered through a 0.45-μm filter to transduce Hep3B cells. DACH1 stable cell pool was characterized using inverted fluorescence microscopy and immunoblotting for their expression levels of DACH1 protein.

### Colony formation assay

In total, 3 × 10^3^ Hep3B cells stably expressing DACH1 or vector control were seeded into 6-well plates in triplicates. Growth medium was exchanged every 3 day. At day 10, cells were fixed with 4% paraformaldehyde for 20mins and stained with 0.5% crystal violet for visualization and counting [38].

### Cell growth and viability assay

To assess the cell growth, 2.5 × 10^3^ Hep3B cells were seeded in 24-well culture plates and the numbers of viable cells were counted every 12 h for three days. Cell viability was determined after 96 h by using the Cell Count Kit-8 (CCK-8) (Dojin Laboratories, Kumamoto, Japan) according to the manufacturer's instruction. Briefly, Hep3B cells were seeded into 96-well plates at an initial density of 2 × 10^3^ cells/well. After 24-, 48-, 72- or 96-h incubation, 10 μl of the kit reagent was added to each well and the plates were incubated further for another 1 h. The absorbance at 450 nm was measured, and the results were plotted as means ± SE.

### Cell migration and invasion assay

For migration assay, Hep3B cells were seeded into six-well plates and the following day multiple scratch wounds were created in the monolayers. In the presence of 0.5% FBS medium, the cells were kept in a humidified incubator at 37°C containing 5% CO_2_. Images were taken at × 100 magnifications at 0, 6, 12, 18 and 24 h and analyzed by Image-Pro Plus 6.0 (Media Cybernetics Inc.). The mean closure of the wound was calculated based on six individual measurements for each wound at each time point. Results were presented as means ± SE.

Invasion assay was performed as described previously with modification [12]. Briefly, 2.5 × 10^4^ Hep3B cells that suspended in DMEM without fetal bovine serum were seeded on an 8-μm pore size Transwell filter insert (Corning Inc., Corning, NY) collocated with Matrigel (BD Biosciences, Bedford, MA), while the lower chamber was filled with medium supplemented with 10% FBS, which acted as chemo-attractants. After incubation for 72 hours at 37°C with 5% CO_2_, the invaded cells were stained with 0.5% crystal violet dissolved in methanol and the number of cells on the bottom were counted under light microscopy (× 200). All experiments were conducted in triplicate.

### Western blot analysis

Cells were seeded in 10 cm dishes (10^6^/well) and collected in RIPA buffer. Thirty micrograms of protein were loaded on 10% SDS-polyacrylamide gels, and the separated proteins were then blotted onto PVDF hybridization transfer membrane. The primary antibodies used were rabbit polyclone DACH1 (ProteinTech group), β-catenin (ProteinTech group), phosph-β-catenin (Santa Cruz), GSK3β (ProteinTech group), phosph-GSK3β (Santa Cruz), cyclin D1 (Cell Signaling Technology), β-actin (Cell Signaling Technology), CDK4 (Santa Cruz), cyclin B1 (Santa Cruz), cyclin E1 (Santa Cruz), PCNA (Santa Cruz) and α-vinculin (Sigma). Secondary staining and detection were conducted according to standard protocols with the enhanced chemiluminescence detection reagent. Results were recorded as fold induction to untreated controls.

### Statistical analysis

The Student's *t*-test and one-way ANOVA were applied to evaluate the differences in groups as appropriate and the significance level was set at 0.05. Correlations between clinicopathological and immunohistochemical variables were calculated according to Person χ^2^ test. Cell culture experiments differences between the groups were evaluated by the Student's *t* test. The cumulative survival time was calculated utilizing the Kaplan-Meier method and analyzed with the log-rank test. Univariate and multivariate analyses were performed based on the Cox proportional hazards regression model. Statistical analyses were conducted using SPSS 12.0 (SPSS, Chicago, IL). Data were presented as mean ± SE.
